# Effective Cytotoxicity of Dendritic Cells against Established T Cell Lymphomas in Mice

**DOI:** 10.4049/jimmunol.2001123

**Published:** 2021-08-15

**Authors:** Sigrid Dubois, Thomas A. Waldmann, Jürgen R. Müller

**Affiliations:** Lymphoid Malignancies Branch, Center for Cancer Research, National Cancer Institute, National Institutes of Health, Bethesda, MD

## Abstract

T cell lymphomas arise in mice that constitutively express a single TCR in the absence of NK cells. Upon TCR engagement these lymphomas are able to corrupt tumor surveillance by decreasing NK cell numbers. In this study, we investigate the outcome of interactions between these T cell lymphomas and dendritic cells. Bone marrow–derived dendritic cells mediated effective killing of T cell lymphomas after activation with IFN-γ and TLR ligands in culture. This cytotoxicity was independent of MHC compatibility. Cell lysis was reduced by the presence of the peroxynitrite inhibitors FeTTPS and L-NMMA, whereas inhibitors of apoptosis, death receptors, and degranulation were without effect, suggesting NO metabolites as the main mediators. When injected together with GM-CSF and R848 into lymphoma-bearing mice, in vitro–expanded bone marrow–derived dendritic cells caused significant survival increases. These data show that dendritic cell adaptive immunotherapy can be used as treatment against T cell lymphomas in mice.

## Key Points

- Stimulated bone marrow–derived dendritic cells lyse T lymphoma target cells in vitro.
- Dendritic cell–mediated cytotoxicity is dependent on peroxynitrite.
- Dendritic cell transfers into T lymphoma-bearing mice show antitumor efficacy.

## Introduction

Mice that constitutively express a single TCR have a low incidence of T cell lymphoma development (1–4). Effective tumor surveillance that is exerted by NK cells inhibits lymphoma development in these mice (5). Engaging the lymphoma TCR in vivo confers the ability to overcome this tumor surveillance by a lymphoma-mediated reduction of NK cell numbers (6). This suggests that TCR stimulation by Ag-presenting dendritic cells (DCs) plays a tumor-promoting role in this lymphoma model (7, 8).

The two main lymphocyte types that are exerting direct cytotoxicity against both solid and hematopoietic tumor targets are CD8 T and NK cells. Killing by CD8 cells is initiated by the presentation of tumor-specific peptides via MHC class I (9). In contrast, NK cells express a plethora of NK cell–specific receptors whose engagement results in activating or inhibiting downstream signaling (10). The recognition of a multitude of ligands for these receptors on tumor targets will cause NK cell cytotoxicity if the balance of receptor signals favors activation. The cytotoxic actions of CD8 and NK cells are somewhat complementary in that CD8 cell action requires MHC class I on the tumor cell surface, whereas low or absent MHC class I constitutes a strong NK cell activator (11).

A third cell type that has been shown to exert direct toxicity toward tumor cells are DCs (12, 13). Although the mechanism of tumor cell recognition remains mainly unknown, DCs clearly distinguish between tumor cells and their nontransformed counterparts. Numerous in vitro data show the ability of various DC subsets, both human and rodent, to lyse large collections of tumor cell types (12, 13). DCs have also been reported to mediate the imiquimod-induced response against basal cell carcinoma (14). Given the primary function of DCs to present Ag, an immunological mechanism termed “killers-gatherers-messengers” has been proposed, in which DCs would phagocytose tumor cells after lysis and subsequently present tumor material to professional killer cells, thus placing DCs into the center of immune response initiation against tumors (12, 13).

In this study, we investigate murine bone marrow–derived DCs as potential effectors to target mouse T cell lymphomas. Our data show a direct cytotoxic response of DCs against T cell lymphomas in vitro and in vivo.

## Materials and Methods

### Reagents

We used LPS (100 ng/ml final concentration in culture, *Escherichia coli* 055:B5; Sigma-Aldrich), PolyI:C (10 μg/ml; Sigma-Aldrich), R848 (1 µg/ml; Invivogen,), CpG (1 µM, ODN 1585; Invivogen,), human IFN-γ (20 ng/ml; PeproTech), murine GM-CSF (4 ng/ml; PeproTech), anti-CD40 (1 µg/ml FGK4.5; BioExpress, Cell Culture Services), Z-VAD-FMK (40 µM; Sigma-Aldrich), FeTTPS (50 µM, purity ≥ 95% by TLC; Calbiochem), L-NMMA (1 mM; Sigma-Aldrich), BAPTA AM (50 ng/ml; Biomol Research Laboratories), concanamycin A (40 ng/ml; Sigma-Aldrich), and blocking Abs against Trail or TNF-α (100 μg/ml; BD Biosciences).

### Cytotoxicity assays and FACS

We generated bone marrow–derived DCs by incubating murine bone marrow for 5 d in RPMI 1640 supplemented with 10% FBS and GM-CSF (seven 20-cm culture plates per femur and tibia). We prepared DCs from bone marrow of C57/BL6, C3H, Ashen, and gld mice. Ashen and C3H mice were provided by Dr. J. A. Hammer, National Heart, Lung, and Blood Institute, National Institutes of Health. Gld mice were purchased from The Jackson Laboratory. For lymphoma-directed cytotoxicity we mixed 7.5 × 10^5^ CFSE-labeled lymphoma cells from spleens of lymphoma-bearing mice or 2.5 × 10^5^ EL4, RMA, or RMA-S–cultured lymphoma cells with 1.5 × 10^5^ DCs (unless otherwise indicated) in 12-well plates, 500 µl per well. RMA and RMA-S cells were kindly provided by Dr. T. Walzer, Le Centre International de Recherche en Infectiologie, Inserm U1111, Lyon, France (15). For cytotoxicity directed against adherent cells, 7.5 × 10^4^ CFSE-labeled B16, MC38, CT26, or TrampC2 tumor cells were seeded 8 h prior to the addition of DCs. Inhibitors were added to targets, as indicated, prior to the addition of DCs. DC stimuli were added 8 h after DCs for most inhibitors; L-NMMA was present for 24 h before the addition of DC stimuli. FACS analyses were done 16 h later to determine the number of surviving tumor cells. For cytometry, cells were harvested, and cultures with adherent targets were filtered through several layers of gauge. Cells were blocked with anti-CD16/32 (15 min at room temperature, clone 93; BioLegend); that was followed by a 30-min incubation on ice with the specific Abs. We used Abs against CD8 (53-6.7; eBioscience) and CD90.1 (HIS51; eBioscience). Data analysis was performed using FlowJo software.

To determine the expression of inducible NO synthase (iNOS), bone marrow–derived DCs were stimulated as above in the absence of tumor cells. Cells were collected and blocked with a mix of rat IgG1, rat IgG2b, rat IgG2a (clones ebrg1, eb149/10H4, ebR2a; eBioscience), and anti-CD16/32 (15 min at room temperature) and stained for CD11b (M1/70; BioLegend) and CD11c (N418; BioLegend). This was followed by fixation in Cytofix/Cytoperm (BD Biosciences) and intracellular staining of iNOS (WT16030C; BioLegend).

### Tumor survival in vivo

For tumor survival studies, we used C57BL/6 mice that were bred in our own animal colony. Animal care and all animal procedures were done in accordance with National Institutes of Health guidelines and were approved by the Animal Care and Use Committee of the National Cancer Institute. For studies with the lymphoma line SJ3 only, mice were depleted of NK cells prior to and during the entire length of the studies by twice-weekly i.p. injections of anti-NK1.1 (25 μg; Bio X Cell). The i.p. tumor transfers were done with SJ3 lymphoma cells (1 × 10^4^ cells per mouse), RMA lymphoma cells (1 × 10^4^) and RMA-S lymphoma cells (1 × 10^5^), whereas B16 melanoma cells (1 × 10^6^) or MC38 colon cancer cells (1 × 10^6^) were injected i.v. We generated syngeneic bone marrow–derived DCs as described above. We removed DCs mechanically from culture plates and injected 1.5 × 10^6^ cells i.p. together with 5 µg of GM-CSF into each mouse 7, 9, and 11 d after tumor transfers for SJ3, MC38, and B16 and 3, 5, and 7 d after transfers for RMA and RMA-S. This was followed by 50 µg of R848 i.p. 3 h after each DC transfer. Because a minority of mice transferred with SJ3 developed fever between 24 and 48 h after the day 7 treatment, all mice received 500 µl of 150 mM NaCl s.c. 8 d after SJ3 tumor transfers. The endpoints were determined by veterinary staff in accordance with objective end point criteria that are specified in the animal protocol.

### Statistical analyses

Analyses were done using Prism 9. Data from cytotoxicity assays were compared using an unpaired *t* test, and two-tailed *p* values are shown. The *p* values for mouse survival were calculated by log-rank (Mantel–Cox) test.

## Results

### Activation-dependent DC cytotoxicity against T cell lymphoma cells in vitro

**FIGURE 1.**
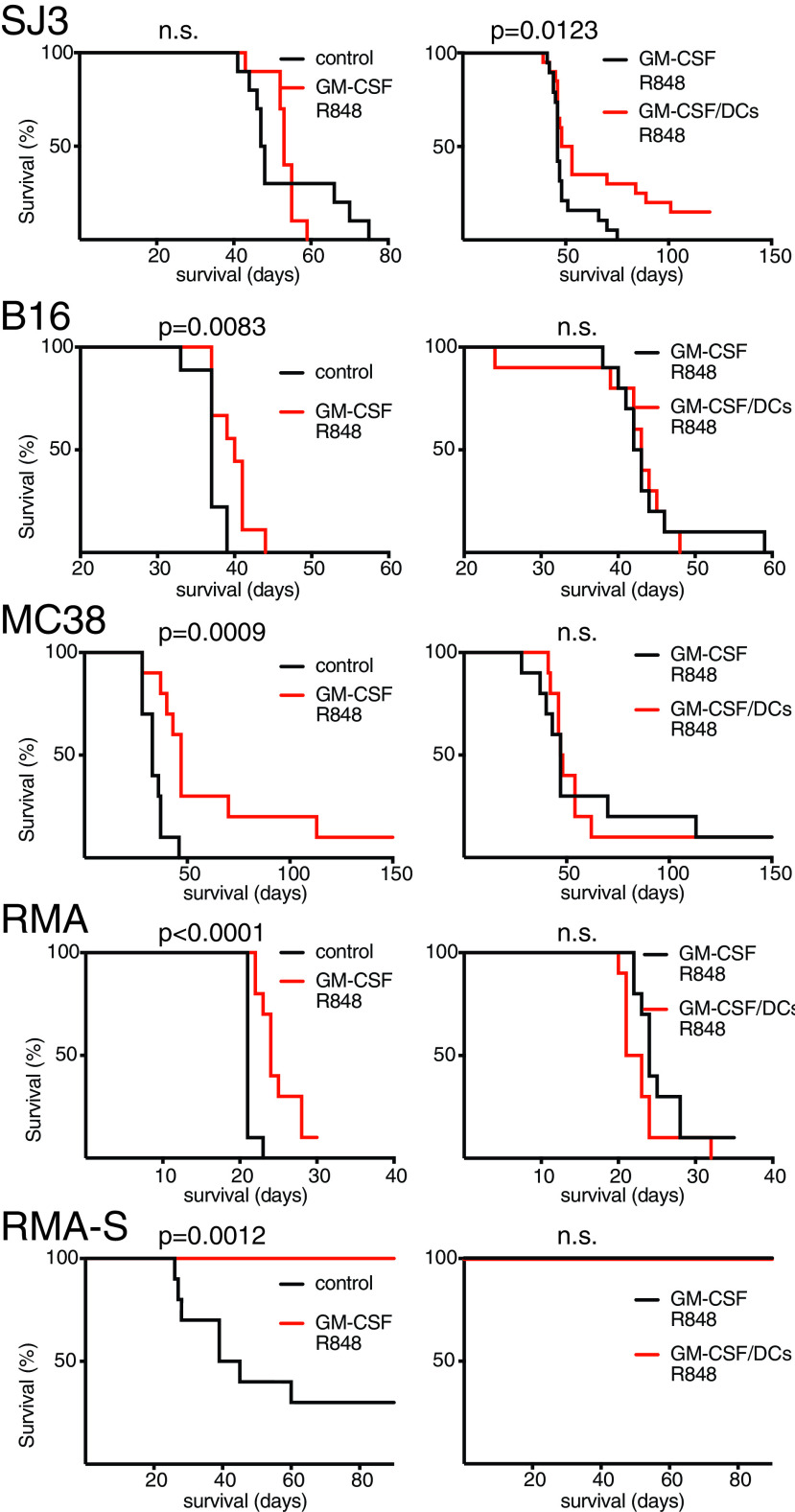
Tumor-directed cytotoxicity of activated DCs. DCs were generated by culturing bone marrow in the presence of GM-CSF, and cytotoxic activities were measured by enumerating the number of surviving tumor cells after overnight coincubations. (**A**) The number of live SJ3 lymphoma cells (rectangle in panels) was unaffected by the presence of either DCs or their activators LPS/IFN-γ, but the combination was associated with a strong reduction of lymphoma cells; representative FACS panels are on top and the left graph below shows averages of five experiments with numbers of surviving SJ3 cells in the absence of both DCs and LPS/IFN-γ set arbitrarily to 100%. The graph also shows that LPS/IFN-γ equally induced DC cytotoxicity against NK cell–resistant SJ3R target cells and against the independently derived SJ4 lymphoma line. *n* = 5 for each bar; conditions without LPS/IFN-γ were set to 100%. Cytotoxic activity could be induced in DCs with TLR ligands, and IFN-γ augmented this activity while being ineffective by itself (middle graph; *n* = 5, condition without DC activation set to 100%). The right graph shows that activated DCs were able to lyse lymphoma target cells at a substantial excess of lymphoma cells; *n* = 5, no DCs = 100%. (**B**) Similar activation patterns were observed with B16 melanoma cells as targets, although IFN-γ showed activity by itself (left graph, *n* = 3). DC activation with LPS/IFN-γ also caused significant cell death in five additional tumor lines (right graph, *n* = 4).

The involvement of DCs in the progression of T cell lymphomas can be both supporting and inhibiting. On one side, DCs provide TCR stimulation that is necessary for lymphoma development (7, 8, 16). Conversely, DC cytotoxicity has the ability to eliminate tumor cells (12, 13). We had recently established T cell lymphoma lines from mice that constitutively express a single TCR in the absence of NK cells. We sought to address the question of DC help versus harm in this lymphoma model by using culture experiments first. We generated murine DCs by culturing bone marrow in GM-CSF and coincubated them with SJ3 lymphoma cells. The presence of DCs, per se, did not appear to affect the number of live lymphoma cells (Fig. 1A, second panel and left graph). However, following maturation with IFN-γ and LPS, DCs had eliminated nearly all SJ3 lymphoma cells in an overnight culture (Fig. 1A, right panel and left graph), whereas IFN-γ and LPS in the absence of DCs did not cause lymphoma death. Similar results were obtained with SJ3 lymphoma cells that had been rendered resistant to NK cells by in vivo TCR engagement [SJ3R (6)] as well as with the independently derived lymphoma line SJ4 [(Fig. 1A, left graph (5)]. Thus, T cell lymphoma cells are targets for the cytotoxic action of activated DCs.

We studied the types of DC activation that causes cytotoxic action against SJ3 lymphoma cells (Fig. 1A, middle graph). All reagents that signal through various TLRs showed efficacy, whereas the engagement of CD40 with an activating Ab appeared to be without effect. IFN-γ itself did not cause DC cytotoxicity but amplified the efficacy of TLR ligands (Fig. 1A, middle graph). We also determined E:T ratios for lymphoma lysis (Fig. 1A, right graph). We observed that substantial cytotoxicity was exerted by DCs to a 10-fold excess of lymphoma cells, thus exceeding the ability of NK cells in similar experiments (5). These data show that an activation via TLR and IFN-γ pathways caused DC-mediated lymphoma lysis in culture.

We determined whether DCs had similar effects on other tumors. Lysis of the melanoma cell line B16 also required DC activation (Fig. 1B, left), although IFN-γ by itself showed efficacy that was not seen with SJ3 lymphoma cells as targets. Overall, when exposed to activated DCs, the numbers of surviving SJ3 lymphoma cells and of B16 melanoma cells were similar (compare (Fig. 1A, middle graph; 1B, left). The two additional T lymphoma lines, EL4 and RMA, as well as three additional lines that had been derived from solid tumors (MC38, TrampC2, and CT26) were also targets of DC-mediated cytotoxicity (Fig. 1B, right). Together, these data show a substantial activation-induced lysis activity of DCs against both solid tumors and T cell lymphomas.

### Lymphoma-targeted DC cytotoxicity is mediated by peroxynitrite

**FIGURE 2.**
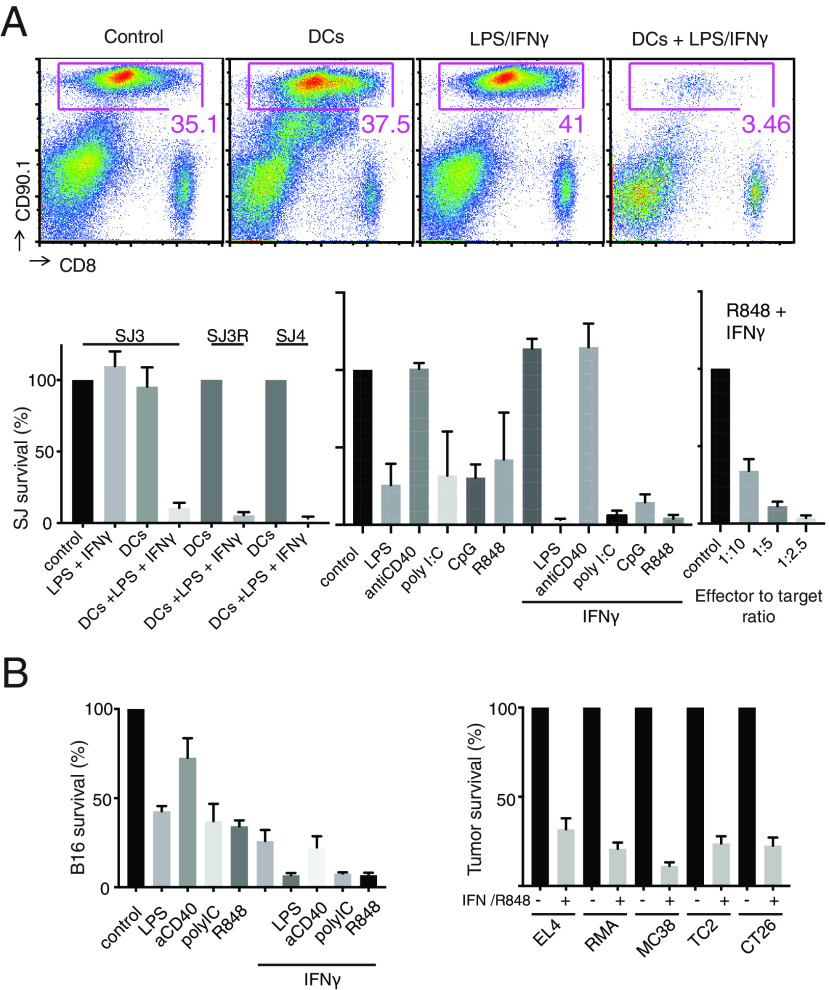
Lymphoma-directed DC cytotoxicity is not MHC restricted and is mediated by peroxynitrite. (**A**) MHC-matched DCs from C57BL/6 mice and nonmatched CH3 DCs were equally able to lyse SJ3 lymphoma cells in the presence of R848 and IFN-γ at the indicated E:T ratios; no DCs condition set to 100%. (**B**) Cytotoxicity does not involve degranulation. Wild-type C3H and degranulation-defective Ashen DCs lysed SJ3 cells equally (control lacks DCs). The presence of the degranulation inhibitors concanamycin A (Conc A) or BAPTA AM (BAPTA) had no significant effect on cytotoxicity (control lacks R848/IFN-γ). (**C**) DC-mediated cytotoxicity of SJ3 is independent of death receptor-induced apoptosis. SJ3 lysis of FasL-defective gld DCs did not differ from wild-type DCs (left, control lacks DCs). Neutralizing Abs against TNF-α and Trail did not prevent lymphoma-directed cytotoxicity (middle, control lacks R848/IFN-γ). Preventing apoptotic cell death by the presence of Z-VAD did not protect lymphoma cells from DC-induced cell death (right, control lacks R848/IFN-γ). (**D**) The presence of the peroxynitrite scavenger FeTTPS significantly reduced DC cytotoxicity toward SJ3 lymphoma cells. Left, Representative FACS panels; the right graph depicts averages ± SD; control lacks R848/IFN-γ. (**E**) Representative FACS panels (top row) and top graph show the induction of iNOS in DCs after activation that is significantly inhibited by L-NMMA. iNOS induction was observed in classical CD11b^+^/CD11c^high^ DCs (bottom three panels). The presence of the iNOS inhibitor L-NMMA also inhibited SJ3 lysis (bottom graph; note logarithmic scale of *y*-axis). *n* = 5 for each condition in this figure. None of the equivalent conditions using different DCs or the presence versus absence of chemical inhibitors other than FeTTPS and L-NMMA were significantly different.

DCs employ various pathways to lyse tumor cells (12, 13). It is commonly described that this cytotoxicity does not require MHC compatibility (17). We investigated this, and we observed that syngeneic and allogeneic DCs that were derived from C57BL/6 and C3H mice, respectively, were equally capable of eliminating SJ3 target cells (Fig. 2A). Because the recognition of targets by cytotoxic CD8 T cells depends on MHC compatibility, this result appears to eliminate the possibility that the cytotoxic activity observed in the presence of DCs is mediated by T cells.

One pathway that has been reported to be employed by cytotoxic DCs involves the degranulation of perforin and granzymes (12, 13). To study this mechanism, we compared the activities of DCs derived from C3H wild-type and from Ashen mice. Ashen mice carry a mutation in Rab27a that prevents degranulation (18). (Fig. 2B (left) shows DC-mediated cytotoxicity regardless of their ability to degranulate. In addition, the presence of the chemical degranulation inhibitors concanamycin A and BAPTA AM (19) failed to prevent cytotoxicity. Thus, degranulation appeared to be without contribution to DC cytotoxicity against lymphoma targets.

The induction of apoptotic cell death by engaging death receptors on target cells via the ligands TNF-α, Trail, and Fas ligand, which are expressed on the surface of DCs, has also been reported (12, 13). We used DCs from gld mice that lack Fas ligand expression (20), and we observed similar lysis capacities when compared with wild-type DCs (Fig. 2C, left). We inhibited the activities of the death receptor ligands Trail and/or TNF-α with neutralizing Abs (19) and observed no effects of the Abs’ presence on cytotoxicity (Fig. 2C, middle). Last, inhibiting apoptosis with Z-VAD also did not alter the outcome of DC-mediated lymphoma lysis. Thus, death receptor ligation does not appear to be necessary to eliminate SJ3 target cells.

Last, the involvement of the NO metabolite peroxynitrite has been previously described (17). Peroxynitrite is scavenged by the hem molecule of erythrocytes, and this can be mimicked by hem derivatives (21–23). We observed that the presence of the peroxynitrite scavenger FeTTPS reduced DC-mediated lymphoma lysis (Fig. 2D). Peroxynitrite generation requires activation of iNOS that can be inhibited by L-NMMA (21–23). We observed that iNOS was present in bone marrow–derived DCs after stimulation with R848/IFN-γ (Fig. 2E, top panels and top graph) and that this induction was slightly but significantly reduced by L-NMMA presence. GM-CSF–induced bone marrow cultures contain a mixture of monocytes and DCs (24). We gated on iNOS-positive cells in these cultures, and we observed iNOS production in classical CD11b^+^/CD11c^high^ DCs, whereas cells that failed to express iNOS after stimulation were lower in CD11c (Fig 2E, lower panels). Preincubations of DCs with the iNOS inhibitor L-NMMA also resulted in a small but significant reduction of cytotoxicity against SJ3 lymphoma cells (Fig 2E, bottom graph). Taken together, these data suggest that the mechanism of lymphoma lysis by activated DCs depends on peroxynitrite but is independent of degranulation, death receptor-induced apoptosis, or of MHC compatibility.

### Efficacy of DC transfers into lymphoma-bearing mice

**FIGURE 3.**
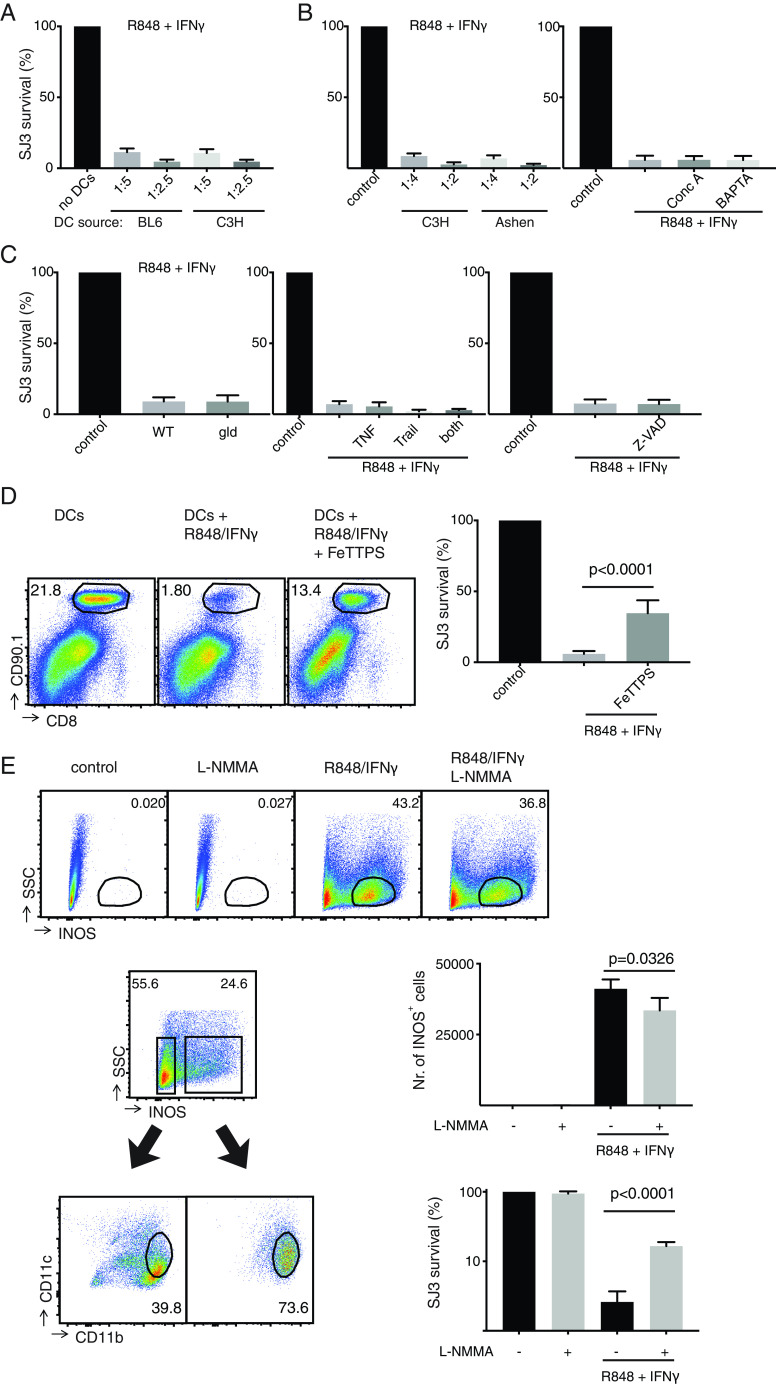
Efficacy of transfers of in vitro–expanded DCs on the survival of SJ3 lymphoma-bearing mice. Mice were implanted with various tumor cells. Treatments done at days 7, 9, and 11 (SJ3, B16, MC38) and at days 3, 5, and 7 (RMA, RMA-S) included the transfers of in vitro–expanded DCs together with GM-CSF and the subsequent injection of the DC activator R848. Treatments with GM-CSF and R848 alone did not significantly affect the survival of SJ3-bearing mice (top row left; *n* = 10 per group), whereas additional DC transfers improved the outcome (right; *p* value above graph; *n* = 20 per group). In all other tumor models, GM-CSF/R848 treatments alone improved outcomes (left graphs), whereas DC transfers showed no additional benefits (*n* = 10 per group). The groups that are depicted in each graph were done in parallel. n.s., not significant.

We studied whether lymphoma-directed cytotoxicity by DCs that we had observed in vitro could be translated to an in vivo setting. We investigated whether DC activation in vivo plus/minus DC transfers would affect the survival of tumor-bearing mice. It has been described that the microenvironment of T cell lymphomas is rich in APCs that are required for lymphoma survival (7, 8). We initially investigated whether the support and activation of DCs in that environment would be sufficient to alter the outcome of the survival of lymphoma-bearing mice. (Fig. 3, top row, left shows that injections of the DC survival factor GM-CSF and the maturation agent R848 alone did not significantly increase the survival of SJ3 lymphoma-bearing mice. Thus, activating endogenous DCs appears to be insufficient to induce measurable anti-lymphoma action.

We next determined effects of DC transfers. We chose a time lapse of 7 d between lymphoma transfers and the beginning of treatments to allow a proper implantation of lymphomas in vivo. DCs were injected together with GM-CSF on days 7, 9, and 11; that was followed by R848 treatments 3 h later. This 3-h delay between DC injection and maturation was done to allow DCs to migrate. (Fig. 3 (top row, right) shows that DC transfers significantly increased the survival of lymphoma-bearing mice. Thus, the transfer of in vitro–expanded DCs into lymphoma-bearing mice appears to improve the response against T cell lymphomas.

We also determined whether DC transfers would improve the survival of mice bearing a variety of other tumor types. We studied two solid tumor models of B16 melanoma and MC38 colon cancer. Both tumors were injected i.v. to induce the formation of lung metastasis, and treatments were started 7 d later. We observed efficacy of GM-CSF/R848 treatments alone leading to medium survival increases from 37 to 40 d and from 33 to 47 d, respectively (Fig. 3, second and third rows, left). This suggests potential effects by endogenous DCs, although further experimentation is required to prove this point. Additional DC transfers, however, did not further improve the outcome (Fig. 3, second and third rows, right). Similar results were obtained with the T lymphoma model RMA (Fig. 3, fourth row). GM-CSF/R848 treatments increased median survival from 21 to 24 d, but no significant effect was achieved with additional DC transfers, suggesting that not all T lymphoma models are affected by adoptive DC transfers. We also tested a derivative of RMA that had been selected for its low MHC class I expression, resulting in RMA-S being targeted by NK cell cytotoxicity (Fig. 3, fifth row (15),). Injections of GM-CSF/R848 alone appeared to have cured all mice, suggesting that DC stimulation may have effects on NK cell activities in this model. In conclusion, these data suggest that transfers of in vitro–expanded DCs may prolong the survival of SJ3 T lymphoma-bearing mice.

## Discussion

DCs appear to play an important role in the life of T cell lymphomas (7, 8, 16). Microenvironments of T cell lymphomas harbor substantial numbers of DCs that provide TCR engagement necessary for the progression of T cell lymphomas. An involvement of Ag-presenting DCs in the survival and progression of T cell lymphomas is also suggested by the relative intactness of TCR signaling cascades in these tumors as well as by the existence of mutations that constitutively activate TCR pathways (7, 8).

The data presented in this paper suggest that this relationship may not always be beneficial for T cell lymphoma survival because DCs appear to have the ability to play a role in the death of these cells also. We show that, at least under in vitro conditions, DCs are transformed into efficient killers of T cell lymphoma cells by an activation with any TLR ligands that is further augmented by IFN-γ presence.

Three classes of mediators of DC-induced tumor cell death have been described: granzymes and perforin that are released by degranulation, death receptor ligands that are induced on the surface of DCs and cause apoptotic death via death receptor ligation on tumor cells, and NO metabolites that are generated after the induction of iNOS (12). The action of death receptor ligands may not require intact DCs but may also be mediated by exosomes (25). The specific use of each class of mediators or combinations thereof are poorly defined but may be affected by the subsets of acting DCs as well as the type of tumor target. We observed that killing the T lymphoma lines did not depend on degranulation or on death receptor ligands but was indeed dependent on peroxynitrite (Fig. 2). These findings are in line with other groups that reported the cytolytic mechanisms of rodent bone marrow–derived DCs to be mediated by NO metabolites (17, 26–29). It is worth noting, in this context, that in our hands all tumor targets tested were sensitive to DC lysis to various degrees and that no distinction between DC lysis-sensitive and -resistant targets could be made.

DCs in the microenvironment of T cell lymphomas appear to be unable to induce significant lymphoma death in response to TLR ligation. The ligation of TLRs is an event that accompanies many conditions from infectious diseases (30) to autoimmune diseases (31), yet T cell lymphomas do not appear to disappear in the cause of such unrelated diseases. In our hands, the systematic application of the TLR7/8 ligand R848 was also inefficient in increasing the survival of SJ3 T cell lymphoma-bearing mice. One explanation is that T cell lymphomas are able to corrupt functions of DCs, turning them, instead, into monocyte-derived suppressor cells and rendering them incapable of exerting their cytotoxic function, as has been described in many tumor models (12, 13). Not all tumor types may have the capacity to inhibit DC functions; a mechanism in basal cell carcinoma has been described in which the topical application of R848 induces a DC response against the tumor, effecting its disappearance (14). We also observed efficacy of GM-CSF/R848 treatments alone in two solid tumor and in another T lymphoma model (Fig. 3). This suggests different levels of immune suppressions that are exerted by different tumors.

The inability to induce antitumor responses by endogenous DCs in the SJ3 model caused us to determine whether a transfer of in vitro–expanded DCs would be more effective to treat T cell lymphomas, and we indeed observed some efficacy, although this effect could not be seen in the other tumor models in which GM-CSF/R848 treatments alone induced antitumor activity (Fig. 3). We also had done a SJ3 survival study that included IFN-γ in addition to R848, but despite its beneficial effects in vitro, IFN-γ inclusion in vivo did not significantly improve the outcome. This may point to additional effects of IFN-γ in vivo that interfere with DC responses and that may not be observed in culture assays with only effector and target cells present.

Our data of DC transfer efficacy appear to support the conclusions from previous reports in which some success with intratumoral injections of immature DCs has been observed (32). The promise of the use of DCs transfers appears obvious; it focuses on another cell type in addition to the professional killers of CD8 and NK cells, thus increasing the diversity of lytic mechanisms as well as the diversity of tumor cell recognition mechanisms (12, 13). Although DCs distinguish between nontransformed and tumor cells (12, 13), recognition mechanisms that underlie these are not understood. Activity of DCs may also be the only choice for immunotherapies of certain tumors, such as the T cell lymphomas in our model, because these cells are able to evade both CD8 T recognition, by downregulating MHC class I, and NK cell activities, by inducing their disappearance (5, 6). In addition, it is believed that DC transfers would cause few side effects (12, 13). We have observed some side effects after transfers in a minority of mice that develop fever symptoms that can be treated with NaCl injections, suggesting that transferred DCs do have effects in vivo.

Another advantage of DC transfers has been described in that DCs can undergo a functional switch from cytotoxic to APCs that culminates in the induction of cytotoxic CD8 or NK cell responses (12, 13), as has been shown in a rat osteosarcoma model (33). Although adoptively transferred DCs may have antitumor activities via stimulating other immune cells rather than by direct cytotoxicity, we do not believe that such a mechanism is involved in the effects in our SJ3 lymphoma model. NK cells have been continuously deleted during the entire course of the in vivo experiments, and CD8 responses appear to be inefficient against these lymphomas, presumably because of low MHC class I expression (5). Indeed, we believe that our data suggest some efficacy of the cytotoxic function of DCs alone after transfer.

Problems with the therapeutic use of DC transfers are equally obvious. The generation of DCs in numbers that are equivalent to the mouse experiments in the range of 1 × 10^10^ cells per treatment appears to be forbiddingly expensive if not impossible with current methodology. Allogenic DCs, although effective in vitro, may have short lifespans in vivo due to MHC incompatibility-induced removal mechanisms. It remains to be seen whether DC culture methods will sufficiently advance to make DC transfer treatments feasible.

In summary, transfers of DCs appear to have some efficacy through cytotoxic activity alone against T cell lymphomas in mice.
